# Harnessing the optimization of enzyme catalytic rates in engineering of metabolic phenotypes

**DOI:** 10.1371/journal.pcbi.1012576

**Published:** 2024-11-04

**Authors:** Zahra Razaghi-Moghadam, Fayaz Soleymani Babadi, Zoran Nikoloski

**Affiliations:** 1 Systems Biology and Mathematical Modeling Group, Max Planck Institute of Molecular Plant Physiology, Potsdam, Germany; 2 Bioinformatics Department, Institute of Biochemistry and Biology, University of Potsdam, Potsdam, Germany; University of Gothenburg: Goteborgs Universitet, SWEDEN

## Abstract

The increasing availability of enzyme turnover number measurements from experiments and of turnover number predictions from deep learning models prompts the use of these enzyme parameters in precise metabolic engineering. Yet, there is no computational approach that allows the prediction of metabolic engineering strategies that rely on the modification of turnover numbers. It is also unclear if modifications of turnover numbers without alterations in the host’s transcriptional regulatory machinery suffice to increase the production of chemicals of interest. Here, we present a constraint-based modeling approach, termed Overcoming Kinetic rate Obstacles (OKO), that uses enzyme-constrained metabolic models to predict *in silico* strategies to increase the production of a given chemical, while ensuring specified cell growth. We demonstrate that the application of OKO to enzyme-constrained metabolic models of *Escherichia coli* and *Saccharomyces cerevisiae* results in strategies that can at least double the production of over 40 compounds with little penalty to growth. Interestingly, we show that the overproduction of compounds of interest does not entail only an increase in the values of turnover numbers. Lastly, we demonstrate that a refinement of OKO, allowing also for manipulation of enzyme abundance, facilitates the usage of the available compendia and deep learning models of turnover numbers in the design of precise metabolic engineering strategies. Our results expand the usage of genome-scale metabolic models toward the identification of targets for protein engineering, allowing their direct usage in the generation of innovative metabolic engineering designs for various biotechnological applications.

## Author summary

Enzymes play a crucial role in metabolic processes, and by selecting enzymes with optimal activity, we can enhance the production of desired compounds. However, there has been no computational approach to designing metabolic engineering strategies based on the modification of enzyme activities. In this study, we developed a new computational method called Overcoming Kinetic rate Obstacles (OKO) to increase chemical production by modifying enzyme activities in *E*. *coli* and *S*. *cerevisiae*. Our method uses growing data on enzyme efficiency from experiments and deep learning models to suggest strategies for metabolic engineering. We applied OKO to increase the production of over 40 different compounds in models of *E*. *coli* and *S*. *cerevisiae*, and found that it can at least double their production without severely affecting cell growth. Finally, we showed that by refining OKO to account for changes in both enzyme activity and abundance, our method can more effectively use existing data and models to design precise strategies for improving chemical production. Our findings pave the way for advanced metabolic engineering techniques for various biotechnological applications.

## Introduction

Microbial cell factories have emerged as a tractable and sustainable solution for the production of important chemicals from renewable biomass [1]. Building such factories necessitates the design and implementation of systems-level metabolic engineering strategies that streamline, modify, and expand the biosynthetic capabilities of microbes serving as design chasses [2]. The *in silico* design of metabolic engineering strategies has been propelled by the developments of constraint-based modeling approaches that make use of genome-scale metabolic models [3–6]. These approaches have focused on *in silico* manipulation of fluxes, achieved *in vivo* by modifying the expression of genes and abundance of downstream proteins by using knock-out, knock-down, or over-expression techniques [7–13]. The production of chemicals by cell factories is, however, often impeded by insufficient catalytic activity of enzymes, resulting in negligible gains despite manipulations in protein abundance. Yet, rational manipulation of the catalytic activity of enzymes has not yet been considered for *in silico* design of metabolic engineering strategies, despite recent advances in enzyme engineering strategies that can be readily employed to address this problem [14].

The turnover number, *k*_*cat*_, of an enzyme denotes the maximum number of substrate molecules converted to product molecules per unit time per active site, and quantifies the enzyme catalytic activity. Recent advances in constraint-based modeling have resulted in approaches that: (i) generate enzyme-constrained genome-scale metabolic models (ecGEMs) [15,16] by integrating turnover numbers from major repositories [17,18] and deep learning predictions [19–22], (ii) combine fluxomics and proteomics data to estimate turnover numbers for several model organisms [23–29], and (iii) correct turnover number estimates to better match measured phenotypes [30,31]. The essence of ecGEMs is that the rate of a metabolic reaction is bounded by the product of the turnover number and abundance of an enzyme catalyzing the reaction. These constraints allow establishing direct relations between metabolic activity, proteome allocation, physiology, and growth for organisms with available ecGEMs, including: *Escherichia coli* [32], *Saccharomyces cerevisiae* [31], Chinese hamster ovary cells [33], *Homo sapiens* [34], *Bacillus subtilis* [35], *Corynebacterium glutamicum* [36], *Chlamydomonas reinhardtii* [28] and *Rhizophagus irregularis* [37]. Importantly, such enzyme constraints indicate a viable way to manipulate native metabolism by designing strategies focused on modification of turnover numbers of enzymes. These strategies can be implemented using advances in deep learning of turnover numbers [19–22], in predictions of protein structures that indicate key amino acid residues driving modifications of catalytic activities [38], and in new tools for directed enzyme evolution [39].

However, the existing ecGEMs have been used to design engineering strategies based only on manipulation of enzyme abundance, following the same principles underlying flux manipulation [7–11]. For instance, an ecGEM of *E*. *coli* was used to improve lysine production, while ensuring fixed minimal growth, by using flux balance analysis (FBA) [40]. Overexpression of proteins with largest predicted abundance was used to validate the predicted increase in lysine production [32]. Similar approach was used with an ecGEM for *C*. *glutamicum* to determine enzymes with predicted differential protein abundance between low and near-optimal growth rate obtained from the *in silico* maximization of lysine production [36]. Another study used an ecGEM of *B*. *subtilis* to identify knockout strategies to overproduce poly-γ-glutamic acid (γ-PGA) [35]. The knock-out strategy was simulated using the well-established minimization of metabolic adjustment method (MOMA) [41], and indicated differences to strategies obtained from the application of MOMA to classical GEMs; the *in vivo* implementation of the resulting strategies indicated a two-fold increase in the production of γ-PGA. While these studies have demonstrated the use of ecGEMs in the design and investigation of strategies to overproduce a few, specific compounds, another study has shown that overproducing certain chemicals including many amino acids, cannot be achieved by manipulation of gene expression due to conflicts arising from promiscuous enzymes, captured by the participation of one gene in the gene-protein-reaction rules of multiple reactions [11]. Specifically, when considering metabolic engineering strategies compatible with gene-protein-reaction rules, present in both ecGEMs and classical GEMs, the flux of one reaction catalysed by a promiscuous enzyme should be increased, while decreasing the flux of another reaction catalysed by the same enzyme. This leads to infeasibilities of designing metabolic engineering strategies that can be resolved by targeted manipulation of turnover numbers that do not tinker with the transcriptional regulatory machinery.

Motivated by developments of ecGEMs that include constraints on how fluxes depend on enzyme abundance and catalytic rates, here we devise, implement, and test constraint-based approaches to design metabolic engineering strategies that: (1) rely on manipulation of turnover numbers, without modifying enzyme abundances compared to the wild type; this avoids the principal problem of gene conflicts arising due to the presence of promiscuous enzymes, (2) consider the simultaneous change of turnover numbers and enzyme abundances, compared to the wild type, and (3) test the resulting strategies by deploying them with a catalogue of proteins with diverse turnover numbers from 343 fungal species. Therefore, we provide a pioneering constraint-based approach applicable with large-scale ecGEMs systematically identifying which enzyme turnover numbers need to be manipulated and in what direction to enhance the capacity of cell factories with respect to a large set of chemicals native to the hosts.

## Results and discussion

### Targeting turnover numbers for engineering applications

The integration of enzymatic constraints into ecGEMs [15] facilitates the development of our optimization-based approach, termed Overcoming Kinetic rate Obstacles (OKO), that predicts metabolic engineering strategies targeting turnover numbers. OKO manipulates the turnover numbers of enzymes under the assumption that the abundances of enzymes in the ecGEMs of wild type and the engineered organism are not significantly changed. This setting is particularly relevant for *in vivo* metabolic engineering strategies, since it does not require tinkering with the underlying transcriptional and (post)translational regulatory machinery of cell factories. That said, practical applications of OKO may still necessitate heterologous expression of genes from other organisms whose proteins have desired catalytic properties.

**Fig 1:**
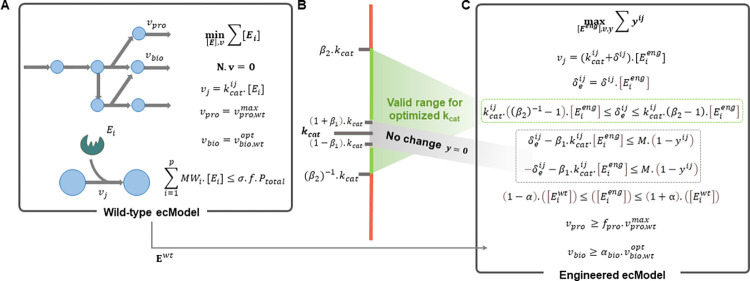
Schematic overview of OKO, a constraint-based approach to predict metabolic engineering strategies targeting turnover numbers. OKO is a two-step approach: In the first step (A), the protein allocation (**E**^***wt***^) is determined by minimizing the enzyme usage at the maximum product yield $\left(v_{pro,wt}^{max}\right)$ in the wild-type model at optimal growth rate ($v_{bio,wt}^{opt}$) under the set of constraints imposed by ecGEMs. (B) It is guaranteed that the changed turnover number is within the admissible range defined by a tuneable parameter ***β***_**2**_. In addition, we introduce a binary variable (***y***) for each turnover number, that keeps track of whether a turnover number is considered to be changed, using a tuneable parameter ***β***_**1**_. The second step of OKO (C) minimizes the number of significantly changed turnover numbers while ensuring that a desired level of chemical production $\left(f_{pro}.v_{pro,wt}^{max}\right)$ is achieved at a factor (***α***_***bio***_) of optimal growth without affecting protein abundance (**E**^***eng***^) compared to the wild type. The small parameter ***α*** quantifies the insignificant deviation from the enzyme abundances in the wild-type model.

OKO is formulated as a two-step approach: In the first step, we determine the maximum product yield in the wild-type model at optimal growth rate under the set of constraints imposed by ecGEMs. Next, we identify the protein allocation by minimizing the enzyme usage (see Fig 1A, Methods). In the second step, with the obtained enzyme abundances in the wild-type model, we constrain the abundance ranges in the model of the engineered strain, to ensure no significant change in comparison to the wild type strain (Methods). By introducing a binary variable for every turnover number, indicating its modification or lack thereof, and a tuneable parameter, specifying the threshold at which changes to the turnover numbers are considered significant, we keep track of whether or not a turnover number is considered changed. Another tuneable parameter is in turn used to define the admissible range for the modified turnover number and guarantees that its value remains within this range (Fig 1B, Methods). The second step of OKO then minimizes the number of significantly changed turnover numbers while ensuring that a desired level of chemical production is achieved at a factor of optimal growth without affecting protein abundance compared to the wild type (Fig 1C, Methods).

### Application of OKO to two cell factories

Modification of enzyme turnover numbers enhances the capacity of wild type cell factories and marks a departure from typical engineering strategies that rely on manipulation of enzyme abundance in prioritizing product formation at the expense of biomass growth. As a result, we expect that by using OKO, the optimal growth rate in in the engineered model may remain almost unchanged in comparison to that of the wild-type, thus breaking the trade-off between product production and growth (or biomass formation) [42]. The reason for this claim lies in the fact that the modification of the turnover numbers, in the absence of optimization of a product of interest, may increase growth in comparison to the wildtype; hence, diverting part of this growth increase towards the production of the chemical of interest can lead to almost unaltered growth while increasing the target production rate compared to the wildtype.

Here, we applied the proposed computational approach, OKO, to the ecYeastGEM_v8.3.4 [31] of the yeast *Saccharomyces cerevisiae* and the eciML1515 [32] of the bacterium *Escherichia coli* as cell factories. We aimed to determine modification strategies that rely on turnover numbers for the overproduction of multiple native compounds in these hosts. The ecYeastGEM v8.3.4 model contains 1148 enzymes, of which 968 are linked to *k*_*cat*_ values, resulting in a total of 4261 available *k*_*cat*_ values that can be modified. The eciML1515 model contains 1505 enzymes with 3392 available *k*_*cat*_ values linked to 1259 enzymes in the model.

Domenzain *et al*. [43] proposed a computational method to predict optimal combinations of gene engineering targets and applied it to the ecYeastGEM_v8.3.4 model to increase the production of 102 different chemicals. Their method involves scoring genes within the model to find the magnitude and directionality of genetic modifications, and then discards genes that encode enzymes that are unfavorable for the production of the target metabolite. Finally, the approach identifies the minimal set of modifications for the transition of cells from biomass formation to metabolic production. Of the chemicals studied in [43], 49 compounds, including amino acids, are native metabolites in *Saccharomyces cerevisiae*. Mapping of these native compounds to metabolites in eciML1515 using KEGG IDs, CHEBI IDs and chemical formulae resulted in 41 metabolites to which the OKO method was applied (see S1 Table).

We first calculated the maximum production of each native compound at optimal growth rates, i.e. 0.38 per hour for ecYeastGEM_v8.3.4 and 0.57 per hour for eciML1515. Enzyme abundances were then calculated by using the first step of OKO (see Methods). To demonstrate that the resulting enzyme abundances can be used as a reference point, we conducted a robustness analysis relying on tailored variability analysis that specifies admissible ranges for enzyme abundance (see Methods). The results show that, on average, the logarithmic ratio of the maximum and minimum values for each enzyme abundance at the maximum production of the different chemicals of interest is 0.015 for the ecYeastGEM_v8.3.4 model and varies from 6×10^−4^ to 0.011 for the eciML1515 model (see S1 and S2 Tables, respectively). This demonstrates that the estimated maximum and minimum abundance values for every enzyme are very close to each other, allowing the usage of the estimated enzyme abundances in the remaining computational steps of OKO. Note that, like in other variability analyses in constraint-based modelling, these estimated ranges are direct consequence of the applied objective and the imposed constraints in the first step of OKO.

We then used OKO to search for strategies with the minimum number of changes in *k*_*cat*_ values that at least double the compound production while maintaining almost the same growth rates as in the wild type. The minimum number of modified *k*_*cat*_ values for overproduction of the chemicals of interest ranges from 7 to 338 for the ecYeastGEM_v8.3.4 model and from 4 to 39 for the eciML1515 model (see S1 and S2 Tables, respectively). The two compounds with the least number of modifications required are choline and palmitoleate in the ecYeastGEM_v8.3.4 model, with seven modified turnover numbers, and proline and serine in the eciML1515 model, with four and five modified turnover numbers, respectively (see S3 Table for the involved enzymes).

Since our goal is to double the production of a chemical of interest in comparison to the wild type, while ensuring certain growth, one may expect that only allowing for an increase in turnover numbers would suffice to address these objectives. Interestingly, however, the increase in production of the considered compounds does not necessarily entail only increases in the turnover numbers. We found that in the ecYeastGEM v8.3.4 model on average 35.5% of the enzymes require an increase in their *k*_*cat*_ values, while in the eciML1515 model, this number is 38.5% to double the production of the selected compounds. Further, since in metabolic engineering it is easier to knock out an enzyme than to reduce its turnover number via enzyme engineering, we also identified the cases where the reduction in turnover number could be replaced by knocking out the enzyme. To the end, we inspected if an enzyme requiring a reduction in *k*_*cat*_ value was associated with a flux of zero (<10^−6^ *mmol gDW*^−1^*h*^−1^) in the engineered model. On average, 53.9% of the reductions in the ecYeastGEM_v8.3.4 model and 12.9% in the eciML1515 model fell into this category. This finding indicated that decrease in turnover number, without blocking of reactions, are required to simultaneously achieve different objectives, including growth and production of the chemical of interest. Indeed, the growth rates of the engineered ecYeastGEM_v8.3.4 and eciML1515 models are on average 88% of the optimal growth rates of the wild-type models, indicating that the increase in the production of compounds following the proposed strategies does come at some, but not drastic cost of growth.

Promiscuous enzymes have been deemed reservoirs of functions that can be used in metabolic engineering, especially if particular catalytic activities are modified in a desired direction [2]. Hence, we also investigated whether enzymes targeted for modification of their turnover number were more likely to be promiscuous. At fixed enzyme abundances, our results imply that the turnover numbers of non-promiscuous enzymes in the model are mostly limiting the over production of the investigated compounds. We found, on average, 26.5% and 24.3% of the enzymes in the proposed strategies for the ecYeastGEM_v8.3.4 and eciML1515 models, respectively, were exhibited promiscuous functions. We further examined the target reactions in the modification strategies to determine whether the estimated turnover numbers suggested by the OKO approach were associated with any promiscuous enzymes for these reactions. In 47% of the proposed modification strategies for the ecYeastGEM_v8.3.4 model and in 62% of the strategies for the eciML1515 model, more than 50% of the estimated turnover numbers were associated with promiscuous enzymes (S1 and S2 Tables).

### OKO identifies strategies for overproduction of amino acids

**Fig 2:**
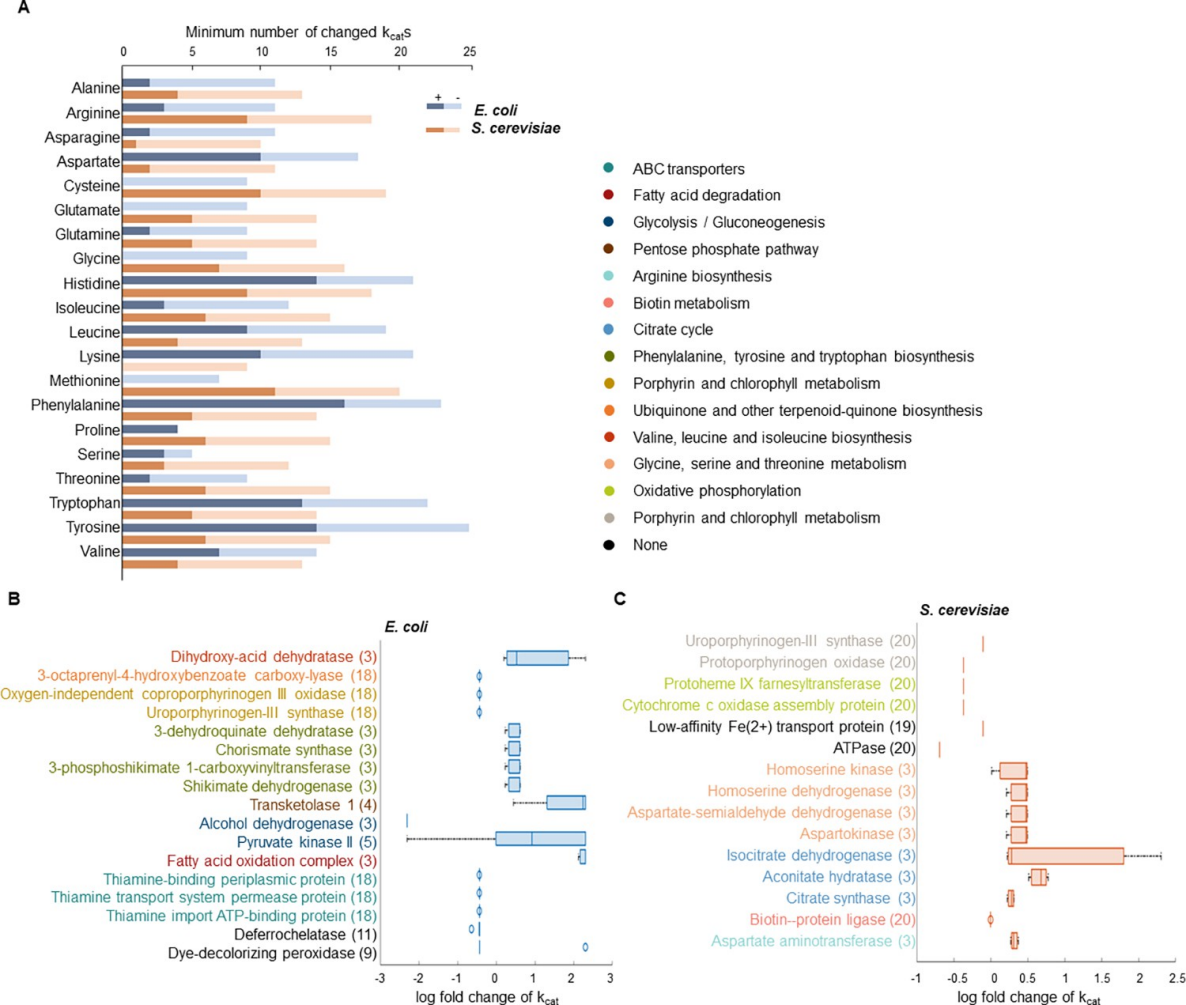
Performance of OKO for amino acids overproduction. (A) The minimum number of modified ***k***_***cat***_s for amino acid overproduction is shown for both *E*. *coli* (blue) and *S*. *cerevisiae* (orange) ecGEMs. Darker (lighter) colors correspond to the number of increased (decreased) ***k***_***cat***_s. In (B) and (C) illustrated are enzymes with modified turnover numbers as part of proposed strategies for overproduction of at least three amino acids in *E*. *coli* and *S*. *cerevisiae* models, respectively. The box plots in each case show the log-fold changes for the enzymes listed in the proposed modification strategies over different amino acids. The numbers in the parentheses indicate the number of modifications for the respective enzymes in the different engineering strategies. The different colors of the enzymes correspond to the biological pathways in which they are involved, described in the legend.

Razaghi-Moghadam et al. [11] investigated the participation of enzymes in different reactions and the inclusion of complex gene-protein-reaction rules, and showed that that the inherent complexity of biological systems is extensively reflected in metabolic models. Therefore, strategies that build on existing enzymes and pathways often lead to conflicts in the manipulation of enzyme abundances. For example, using their proposed approach, it was found that there is no feasible strategy at the native enzyme level to increase production of almost all amino acids in *E*. *coli* and *S*. *cerevisiae*. This finding motivated us to further investigate the performance of OKO to increase the production of amino acids in these cell factories. To this end, we first reviewed the primary results of OKO, which indicated the minimum number of modified turnover numbers for amino acid overproduction. This number ranges from 9 to 20 for the ecYeastGEM_v8.3.4 model and from 4 to 25 for the eciML1515 model (Fig 2A). We found that, on average, 35% and 37.4% of the proposed enzymes in the ecYeastGEM_v8.3.4 and eciML1515 models, respectively, required an increase in their *k*_*cat*_ values.

Furthermore, we observed that the proposed amino acid modification strategies involve a total of 83 enzymes in the ecYeastGEM v8.3.4 model and 80 enzymes in the eciML1515 model. Of these, 15 enzymes in the ecYeastGEM v8.3.4 model and 17 enzymes in the eciML1515 model are required for the overproduction of at least three amino acids. These enzyme names are illustrated in Fig 2B and 2C, where the number of amino acids for which each enzyme modification is required and the log-fold changes of the enzymes are visually depicted. Moreover, and most importantly, the relative changes in the *k*_*cat*_ values are small, reaching the defined limits (either (*β*_2_)^−1^ or *β*_2_) only 0.6% and 7.6% of the time for the ecYeastGEM v8.3.4 and eciML1515 models, respectively (see S4 and S5 Tables).

In addition, as shown in Fig 2B and 2C, for each enzyme included in the proposed amino acid modification strategies, the log-fold changes are predominantly distributed to one side of zero, indicating that the proposed modifications are biased towards either increasing or decreasing the *k*_*cat*_ values to increase the production of each amino acid. Furthermore, the boxplots in Fig 2B and 2C show a small spread, indicating the uniformity of the estimated *k*_*cat*_ values for each enzyme. Therefore, *in vivo* enzyme engineering following the proposed strategy for overproduction of amino acids is expected to benefit the increase of production of other amino acids that also relies on that enzyme.

As depicted in Fig 2B and S4 Table, the eciML1515 model demonstrates two exceptions to the consistency of change directions. The *k*_*cat*_ value of dye-decolorizing peroxidase YfeX enzyme (Uniprot ID P76536) in the ferrochelatase reaction is initially 25999.42 (s^−1^) in the eciML1515 model. For the overproduction of alanine, aspartate, cysteine, glutamine, glycine, threonine, tryptophan, and tyrosine, this value must be reduced by a factor of 0.7. Conversely, overproduction of proline requires a 10-fold increase in the *k*_*cat*_ value. In addition, pyruvate kinase II (Uniprot ID P21599) with an initial *k*_*cat*_ value of 3204.01 (s^−1^) in various pyruvate kinase reactions of the eciML1515 model needs to be increased for arginine and isoleucine overproduction and decreased for tyrosine overproduction. In the proposed strategy for arginine overproduction, the *k*_*cat*_ value of pyruvate kinase II needs to be increased 10-fold in two reactions and 2.5-fold in another reaction.

In the ecYeastGEM v8.3.4 model, as shown in Fig 2C and detailed in S5 Table, the estimated changes in *k*_*cat*_ values for two enzymes show greater variability. The isocitrate dehydrogenase [NADP] cytoplasmic (IDH) enzyme (Uniprot ID P41939) has a k_cat_ value of 58.30 (s^−1^) in the isocitrate dehydrogenase (NADP) reaction. This value needs to be increased by a factor of 1.3 for the overproduction of glutamine and proline, and by a factor of 10 for the overproduction of glutamate. In addition, the homoserine kinase (HK) (HSK) enzyme (Uniprot ID P17423), which has a k_cat_ value of 40.80 (s^−1^) in the homoserine kinase reaction, needs to be increased 1.6-fold for glycine and threonine overproduction, whereas only a 1.01-fold increase is suggested for isoleucine overproduction. These examples demonstrate that while changes in some turnover numbers can generally accommodate usage of the engineered enzymes in the implementation of engineering strategies for different compounds, there are few, notable exceptions.

As discussed above, the decrease in turnover number is required to simultaneously achieve different objectives, including growth and production of the chemical of interest. We further examined the enzymes for which a reduction in the turnover number was proposed to increase the production of at least one amino acid (see S6 and S7 Tables). In the case of ecYeastGEM_v8.3.4, there were eight different enzymes of this type involved in *porphyrin and chlorophyll metabolism*, *oxidative phosphorylation* and *biotin metabolism*. For eciML1515, 13 enzymes were targeted for reduction in turnover number, mainly involved in *porphyrin and chlorophyll metabolism*, *ubiquinone and other terpenoid-quinone biosynthesis* and *ABC transporters*.

This study presents the first *in silico* optimization method targeting enzyme activity rather than flux or enzyme abundance. Consequently, direct comparisons with existing approaches may not be entirely appropriate. Nonetheless, to check the extent to which the manipulation of the catalytic activity of enzymes improves the proposed metabolic engineering strategies, we compared the strategies proposed by OKO with those of OptReg [8], an alternative approach that only focuses on *in silico* manipulation of fluxes (see S8 Table). The results showed that, on average, in ecYeatGEM_v8.3.4 and eciML1515, the number of required manipulations proposed by OptReg was 12 and 17.22 times higher than those proposed by OKO, respectively.

### Setting the ground for experimental validation

Metabolic engineering approaches that aim to use enzymes with the optimized *k*_*cat*_ values can leverage either existing nonnative enzymes or engineered enzymes with modified substrate specificity. Methods that rely on existing enzymes have the flexibility to reuse the enzyme repository in nature and facilitate the creation of synthetic pathways for engineering purposes. This motivated us to investigate whether the identified *k*_*cat*_ values in our engineering strategies are close to the turnover numbers of these enzymes in other organisms.

Enzyme databases such as BRENDA [17] and SABIO-RK [18] contain extensive repositories of *k*_*cat*_ values. However, there is still a lack of high-throughput techniques for measuring *k*_*cat*_ values, resulting in a notable disparity in the number of *k*_*cat*_ values available compared to the vast number of organisms and metabolic enzymes in existence [29]. To fill this gap, deep learning and machine learning approaches have been proposed to predict the *k*_*cat*_ values for different enzymes in different organisms, and have shown high performance [19–22]. To investigate whether the *k*_*cat*_ values estimated by OKO could be found for the respective enzymes in other organisms, we used the predicted *k*_*cat*_ values from the DLKcat [19] and TurNup [20] approaches. DLKcat includes the prediction of *k*_*cat*_ values for 651 metabolic enzymes in 343 organisms (see S9 Table). We employed another deep learning method, TurNup [20], to predict turnover values for the same 343 organisms for which turnover numbers were predicted using the DLKcat. Altogether, TurNup includes the prediction of *k*_*cat*_ values for 630 metabolic enzymes in 343 organisms (see S10 Table).

Having mapped the two sets of *k*_*cat*_ values from ecGEMs and the DLKcat results (see Methods), we examined each enzyme with an estimated *k*_*cat*_ value from OKO to check if it was included in the list of enzymes with a predicted *k*_*cat*_ value from the DLKcat approach. If an enzyme was included, we then identified the closest *k*_*cat*_ value to the estimated one across all organisms. In the proposed amino acid modification strategies, we identified 248 and 236 estimated *k*_*cat*_ values for the ecYeastGEM_v8.3.4 and eciML1515 models, corresponding to 83 and 80 different enzymes, respectively. As shown in Table 1, only 55.2% and 24.2% of these enzymes were included in the list of enzymes with predicted *k*_*cat*_ values by the DLKcat approach. Additionally, the average distance of the closest *k*_*cat*_ values to the estimated values across all organisms was 503.15 (*s*^−1^) and 32188.07 (*s*^−1^) for the ecYeastGEM_v8.3.4 and eciML1515 models, respectively. These findings indicated that the enzymes with *k*_*cat*_ values estimated by the OKO approach were rarely found in other organisms and could not be directly used for *in vivo* engineering. Specifically, these results show that the OKO approach is effective in identifying optimal kinetic parameters that may not occur naturally, demonstrating its strength in predicting novel enzyme functionalities. However, it also highlights the challenge of translating these theoretical predictions into practical applications, requiring the design or engineering of new enzymes to achieve the desired catalytic efficiency *in vivo*. This highlights the need to impose constraints on the estimated turnover numbers, which we address in the following section by detailing the methodology used for these constraints and assessing their impact on the results.

**Table 1:** Deep-learning-driven identification of suitable turnover numbers from other organisms. Shown is the number of enzyme-reaction pairs in the proposed strategies for which turnover numbers can be predicted by DLKcat and TurNup. The table also includes the average distance of the *k*_*cat*_ values predicted by DLKcat and TurNup and the modifications resulting from OKO and OKO^+^, respectively.

	OKO		OKO^+^ (using DLKcat)		OKO^+^ (using TurNup)	
	*E*. *coli*	*S*. *cerevisiae*	*E*. *coli*	*S*. *cerevisiae*	*E*. *coli*	*S*. *cerevisiae*
Found enzyme-reaction pairs	57	137	354	817	365	1559
Missing enzyme-reaction pairs	179	111	0	0	0	0
Average distance (*s*^−1^)	32188.07	503.15	2.35	4.92	61.31	346.62

### Refinement of OKO enhances its applicability

As it is more convenient to construct synthetic pathways using existing enzymes, we also proposed a refinement of OKO, termed OKO^+^. In OKO^+^, for each enzyme-reaction pair with an available *k*_*cat*_ value in an ecGEM, we first extract the minimum and maximum of the predicted *k*_*cat*_ values in all other organisms using the mapping established between ecGEM and DLKcat results. In total, there are 4261 and 3392 enzyme-reaction pairs with available *k*_*cat*_ values in the ecYeastGEM_v8.3.4 and eciML1515 models, respectively, of which 1676 and 469 respected values are found in the predicted *k*_*cat*_ values from the DLKcat approach (see S11 Table). We introduce two additional sets of constraints in OKO^+^ to ensure that only enzymes with found predicted turnover numbers can be targeted: (1) for the enzyme-reaction pairs with available turnover predictions from DLKcat or other resources, the predicted *k*_*cat*_ should remain either unchanged or within the range extracted from the values obtained from DLKcat, and (2) the enzyme-reaction pairs with available *k*_*cat*_ values in the ecGEM, for which values from DLKcat (from other organisms) are not available, are not allowed to be modified.

The introduction of these two additional sets of constraints resulted in infeasibilities to identify engineering strategies that yield the desired levels of product formation and growth. Therefore, in OKO^+^, we also allow for variation in enzyme abundance, modelled by introduction of a binary variable for each enzyme abundance indicating if it is significantly altered (with respect to a tunable parameter). This approach then minimizes the weighted average of the number of modified turnover numbers and the number of modified abundances, with preference for the former (see Methods).

By deploying OKO^+^ with the same models we found that the minimum number of modified *k*_*cat*_ values for overproduction of the chemicals of interest ranges from 0 to 85 for the ecYeastGEM_v8.3.4 model and from 0 to 44 for the eciML1515 model (see S1 and S2 Tables), while also requiring changes in enzyme abundance. The ecYeastGEM_v8.3.4 model requires different number of changes in enzyme abundance, ranging from 2 to 240 modifications, while this number varies from 3 to 286 for the eciML1515 model. On average, 48.8% and 77% of the enzymes in the proposed strategies for the ecYeastGEM_v8.3.4 and eciML1515 models were promiscuous enzymes, respectively.

In addition, for each *k*_*cat*_ value estimated by OKO^+^ in amino acid modification strategies, we identified the closest *k*_*cat*_ value across all organisms. Clearly, due to the constraints imposed in OKO^+^, all enzymes with newly estimated *k*_*cat*_ values were included in the list of enzymes with predicted *k*_*cat*_ values from the DLKcat approach. The average distance of the estimated values to their closest counterparts across all organisms was 2.35 for the ecYeastGEM_v8.3.4 model and 4.92 for the eciML1515 model (see Table 1). The small average distance value indicates that OKO^+^ successfully identified a suitable enzyme for another organism with closely matching activity.

Inspection of the proposed strategies to increase the production of amino acids showed that the existing nonnative enzymes with *k*_*cat*_ values closest to the predicted by OKO^+^ originate from different organisms (see S1, S2 and S12 Tables). In total, 103 different organisms contribute to the proposed modification strategies for the ecYeastGEM_v8.3.4 model and 53 for the eciML1515 model (see S12 Table). Each organism contributes a different number of enzymes, ranging from 1 to 50 for the proposed modifications in the ecYeastGEM_v8.3.4 model and from 1 to 29 for the eciML1515 model.

To further analyze the applicability of the strategies resulting from OKO^+^, we constructed two distinct ecGEMs by substituting the *k*_*cat*_ values in the original ecGEMs with the values estimated by OKO^+^, as well as the closest values from other organisms. We then assessed the performance of the strategies with the so-modified turnover numbers in producing the compounds of interest. In each model constructed and for each compound of interest, the biomass level was set to match that of the proposed engineering strategy for the overproducing that chemical. In addition, the number of modifications to the enzyme abundance was constrained by the number of modifications in the corresponding proposed engineering strategy. Clearly, this evaluation is only applicable in cases where at least one modification to the enzyme activity was proposed by OKO^+^ and this is for 19 amino acids in the ecYeastGEM_v8.3.4 model and 12 in the eciML1515 model. With these constraints, we calculated the maximum production of each chemical of interest in the two constructed ecGEMs and determined their ratio $\left(\frac{v_{max}^{DLKcat}}{v_{max}^{{OKO}^{+}}}\right)$ (see S1 and S2 Tables). In the majority of cases the two constructed ecGEMs yielded the same levels of product formation. Small differences in product formation levels were only observed for arginine in the eciML1515 model, with the ratio being 0.97.

### Effect of deep learning model for turnover numbers on predicted engineering strategies

To evaluate the impact of deep and machine learning methods on the engineering strategies proposed by OKO^+^, we employed another deep learning method, TurNup [20], to predict turnover values for the same 343 organisms used with DLKcat. We used the predicted values to constrain the optimization approach. TurNup predicted 2912 and 943 turnover numbers for enzyme-reaction pairs with available *k*_*cat*_ values in the ecYeastGEM_v8.3.4 and eciML1515 models, respectively (see S13 Table).

According to the constraints in OKO+, the higher number of predicted turnover numbers from TurNup compared to DLKcat should theoretically provide more opportunities for modification, thereby reducing the number of changes needed. However, in the case of ecYeastGEM_v8.3.4, out of 1470 shared enzyme-reaction pairs from both approaches, 854 pairs have ranges of change from the TurNup approach that fall within the ranges from the DLKcat approach. This can result in fewer opportunities for modification and an increase in the number of proposed changes (see S1 and S2 Tables). Similarly, for eciML1515, there are 469 shared enzyme-reaction pairs between the two approaches, of which 282 pairs have ranges of change from the TurNup approach that are encompassed by the ranges from the DLKcat approach. This indicates that it is not reasonable to compare the results of OKO^+^ using different prediction models, and highlights the need for more refined prediction models to effectively improve optimization strategies.

The average difference of the estimated values to their closest counterparts across all organisms was 346.62 (s^−1^) for the ecYeastGEM_v8.3.4 model and 61.31 (s^−1^) for the eciML1515 model (see Table 1). The average distance values indicate that OKO^+^ identified closer enzymes from another organism with matching activity using the list of predicted turnover numbers from DLKcat compared to that from TurNup. In total, using the predicted turnover numbers by TurNup, 203 different organisms contribute to the proposed modification strategies for the ecYeastGEM_v8.3.4 model and 33 for the eciML1515 model (see S14 Table). Each organism contributes a different number of enzymes, ranging from 1 to 86 for the proposed modifications in the ecYeastGEM_v8.3.4 model and from 1 to 27 for the eciML1515 model.

### Feasibility of strategies designed by employing OKO^+^

**Fig 3:**
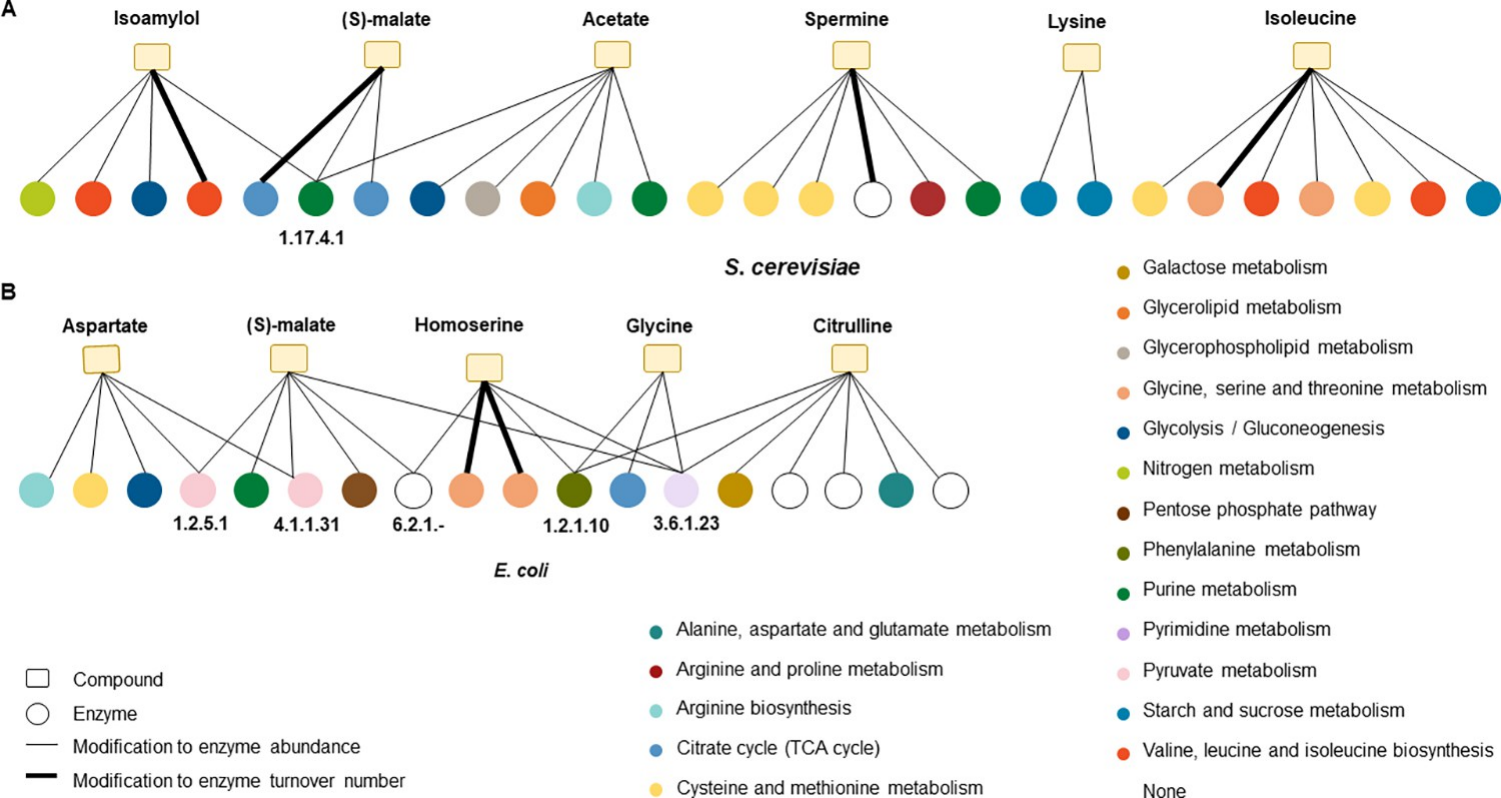
Proposed metabolic engineering strategies presented as bipartite graphs. Bipartite graphs are constructed to represent associations between enzymes to be modified for engineering purposes and compounds for which the OKO+ engineering strategies involve at most seven modifications. Six such compounds were identified in the *S*. *cerevisiae* model (A) and five in the *E*. *coli* model (B). The EC numbers are provided only for those enzymes to which modifications contribute to the overproduction of more than one compound. Thicker edges are associated with modifications to the enzyme turnover numbers. The different colors of the enzymes correspond to the biological pathways in which they are involved.

OKO^+^ was employed to design engineering strategies for the overproduction of 49 compounds in the ecYeastGEM_v8.3.4 model and 41 compounds in the eciML1515 model. The total number of modifications, including enzyme abundance and turnover numbers, varied from 2 to 293 in the ecYeastGEM_v8.3.4 model and from 3 to 316 in the eciML1515 model (see S1 and S2 Tables). Further analysis focused on the engineering strategies of OKO^+^ that are more feasible to implement, specifically those with a total number of modifications less than or equal to seven. Six such compounds were identified in the ecYeastGEM_v8.3.4 model and five in the eciML1515 model (see Table 2 and Fig 3).

**Table 2:** Proposed metabolic engineering strategies with feasible implementation. Shown are the compounds for which the proposed engineering strategies involve a total of seven or fewer modifications. The number of modifications to the turnover number and to the enzyme abundance are included.

		modifications of turnover number	Modifications of enzyme abundance
***E*. *coli***	Aspartate	0	5
	Citrulline	0	7
	Glycine	0	3
	Homoserine	2	3
	(S)- malate	0	6
	Acetate	0	6
***S*. *cerevisiae***	Isoamylol	1	4
	Isoleucine	1	6
	Lysine	0	2
	Spermine	1	5
	(S)-malate	1	2

It is noteworthy that all modification strategies proposed by OKO^+^ to overproduce these eleven compounds involved an increase in enzyme abundance. Furthermore, the proposed engineering strategies do not only involve modification of enzyme abundance, but also adjustment of enzyme turnover numbers. For instance, the overproduction of isoamylol requires a 1.4-fold increase in the k_cat_ value of the 3-isopropylmalate dehydrogenase enzyme (Uniprot ID P04173), with the closest comparable value found in *Candida rhagii*. Similarly, for the overproduction of isoleucine, a 1.2-fold increase in the k_cat_ value of homoserine dehydrogenase (Uniprot ID P31116) is required, and the closest identified value comes from *Blastobotrys raffinofermentans*.

In the proposed strategy for spermine overproduction, a 1.5×10^−4^-fold reduction in the *k*_*cat*_ value of fumarate reductase 2 (Uniprot ID P21375) was proposed, with the closest corresponding value identified in *Ogataea trehaloabstinens*. Finally, for (S)-malate overproduction, the proposed strategy involved a 1.6-fold increase in the k_cat_ value of pyruvate carboxylase 1 (Uniprot ID P11154), with the closest comparable value coming from *Lachancea waltii*.

Additionally, for increasing homoserine production in the eciML1515 model, OKO^+^ proposed an engineering strategy involving a 1.3-fold increase in the k_cat_ value of aspartate-semialdehyde dehydrogenase (Uniprot ID P0A9Q9) and a 2.9-fold increase in the k_cat_ value of the bifunctional aspartokinase/homoserine dehydrogenase 1 (Uniprot ID P00561). The closest comparable *k*_*cat*_ values to these proposed modifications are identified in *Saturnispora zaruensis* and *B*. *anomalus*, respectively. These results jointly indicate that feasible engineering strategies can be obtained when combining overexpression of enzymes with enzymes engineered towards an increase in turnover numbers.

## Conclusions

Availability of enzyme turnover numbers, estimated by coupling omics data with models or relying and classical experimental techniques or predicted by machine and deep learning approaches, holds the promise for their usage in the design and deployment of precise metabolic engineering strategies. However, despite this promise, metabolic engineering has not capitalized from the availability of repositories of and deep learning models for turnover numbers. We address this gap by proposing, implementing, and testing a constraint-based approach, termed OKO, for the design of metabolic engineering strategies that identifies which turnover numbers need to be modified alongside the extent of the change. In experimental setting, the identified modifications can be achieved by using either the two well-established strategies for protein engineering, i.e. rational protein design and directed evolution [44], or relying on proteins from other organisms for which turnover numbers can be predicted by deep learning models [19–22]. In the first case, one can make use of advances in CRISPR-Cas9 system for precise single-base mutations (and combinations thereof) in combination with the pocketome databases [45] to identify active site residues that lead to the proposed increased or decreased catalytic rates of a protein for a given reaction. In the second case, one can knock-out the promiscuous enzyme in the host and make use of heterologous non-promiscuous enzymes for the reactions of interest, if such can be identified with computational search (as done in our study, relying on models for prediction of catalytic rates). Despite the existence of these options, further advances in structural biology and synthetic biology will be needed to overcome challenges in implementing the proposed strategies.

Our findings indicated that the use of OKO with enzyme-constrained metabolic models of *E*. *coli* and *S*. *cerevisiae* resulted in metabolic engineering strategies that overcome the trade-off between growth and increased production of a chemical of interest. Importantly, we found that the overproduction of chemicals of interest does not only entail an increase in selected enzymes. Motivated by the need to consider the available estimates and predictions of turnover numbers for selected enzymes, we also presented a refinement of OKO that combines the modification of turnover numbers (from a well-defined range) with alterations of enzyme abundances. The resulting approach resulted in feasible strategies for overproduction of amino acids in the two hosts, which was not possible to obtain with other constrained-based approaches focused only on the alteration of gene expression (as a proxy for enzyme abundance). However, our findings indicated that the metabolic engineering strategies strongly depend on the predictions of the deep learning models for turnover numbers. It is important to note that the effectiveness of the proposed strategies also depends on the accuracy of the initial turnover numbers used in the model. Altogether, our study paves the way for the deployment of precise metabolic engineering strategies that rely on the natural diversity of turnover numbers for given enzymes or that can use techniques for enzyme engineering.

## Methods

### Formulation of OKO

To determine the abundance of enzymes at the maximum product yield ($v_{pro,wt}^{max}$) in the wild-type model, while ensuring optimal growth rate ($v_{bio,wt}^{opt}$), we solve the following linear programming problem (LP1):

$$\underset{v,E}{\min}\sum_{i=1}^{p}\left[E_{i}\right]$$ (1)

s.t

N∙v=0 (2)

$$v_{j}^{min}\leq v_{j}\leq v_{j}^{max},1\leq j\leq r$$ (3)

$$v_{pro}\geq 0.98∙v_{pro,wt}^{max}$$ (4)

$$v_{bio}\geq 0.98∙v_{bio,wt}^{opt}$$ (5)

$$v_{j}=k_{cat}^{ij}∙[E_{i}],1\leq j\leq r,1\leq i\leq p$$ (6)

$$\sum_{i=1}^{p}{MW}_{i}∙{[E}_{i}]\leq \sigma ∙f∙P_{total}$$ (7)

where [*E*_*i*_] is the abundance of enzyme *i*, *p* denotes the number of enzymes, **N** is the stoichiometric matrix, **v** is the flux distribution vector, *v*_*j*_ is the metabolic flux through reaction *j*, *r* is the number of reactions, *v*_*pro*_ and *v*_*bio*_ are the fluxes through the synthesis of the product of interest and the biomass reaction, respectively, $k_{cat}^{ij}$ is the catalytic rate of enzyme *i* catalysing reaction *j*, *MW*_*i*_ is the molecular weight of enzyme *i*, *σ* is the average *in vivo* enzyme saturation, *f* is the mass fraction of all measured enzymes included in the model, and *P*_*total*_ is the total protein content.

We use the resulting obtained enzyme abundances ($\left[E_{i}^{wt}\right]$) to constrain the abundance range in the engineered model, using the following constraint:

$$(1-\alpha )∙(\left[E_{i}^{wt}\right])\leq [E_{i}^{eng}]\leq (1+\alpha )∙(\left[E_{i}^{wt}\right]),1\leq i\leq p$$ (8)

where *α* is a small parameter (≈10^−1^) that quantifies the insignificant deviation from the enzyme abundances in the wild-type model. Therefore, for $\left[E_{i}^{eng}\right]$, we still allow a deviation from $\left[E_{i}^{wt}\right]$, but not a large one, as controlled by the parameter α.

To overcome the shortcomings of abundance-based strategies [7–11], OKO opt to develop strategies that manipulate the turnover numbers. Accordingly, in the engineered model, the constraint in Eq (6) is replaced by the following constraint:

$$v_{j}={(k}_{cat}^{ij}+\delta^{ij})∙[E_{i}^{eng}],1\leq j\leq r,1\leq i\leq p$$ (9)

where *δ*^*ij*^ shows the deviation from the turnover number of the wild-type enzyme, and it can be positive or negative. Eq (9) contains a bi-linear term which includes *δ*^*ij*^ and $\left[E_{i}^{eng}\right]$. The Petersen linearization scheme, previously used in metabolic modeling [46], is applicable when one variable is binary, which is not the case here. Therefore, here to linearize this constraint a new variable $\delta_{e}^{ij}=\delta^{ij}.[E_{i}^{eng}]$ and the following constraints are added to the optimization problem:

$$v_{j}=k_{cat}^{ij}∙[E_{i}^{eng}]+\delta_{e}^{ij},1\leq j\leq r,1\leq i\leq p$$ (9’)

$$-M∙[E_{i}^{eng}]\leq \delta_{e}^{ij}\leq M∙[E_{i}^{eng}],1\leq j\leq r,1\leq i\leq p$$ (10)

where *M* is a large number (10^6^) chosen to be greater than all upper bound [46], and Eq (10) guaranties that $[E_{i}^{eng}]=0$ implies $\delta_{e}^{ij}=0$. As a result, Eq (9) is replaced by Eq (9’) to remove the bi-linear constraint. The optimized turnover numbers can then be calculated by kcatij*=kcatij+δij=kcatij+δeij[Eieng]. For every $k_{cat}^{ij}$ value, a binary variable *y*^*ij*^ is introduced; it takes the value of 0, if the catalytic rate is significantly changed, and 1, otherwise. For each $k_{cat}^{ij}$, we also define two intervals (see Fig 1B) within which the optimized value may fall. These intervals are adjusted to force the optimized value to be significantly, but not excessively away from $k_{cat}^{ij}$: if $(1-\beta_{1})∙k_{cat}^{ij}\leq k_{cat}^{ij*}\leq (1+\beta_{1})∙k_{cat}^{ij}$, then we consider the value of $k_{cat}^{ij}$ to not have significantly changed and *y*^*ij*^ equals 1. Further, we prevent large relative changes by ensuring that ${\left(\beta_{2}\right)}^{-1}∙k_{cat}^{ij}\leq k_{cat}^{ij*}\leq \beta_{2}∙k_{cat}^{ij}$. The two tuneable parameters *β*_1_ and *β*_2_ define the allowed intervals for the optimized catalytic rate ($k_{cat}^{ij*}$), i.e. *β*_1_(≈10^−8^) specifies the initial threshold for what is considered a change and *β*_2_(≈10) sets the upper limit for acceptable change. Based on the following constraints, the binary variable *y*^*ij*^ enables us to switch between intervals and determine whether the $k_{cat}^{ij}$ can be changed or not:

$${-\delta }_{e}^{ij}-\beta_{1}∙k_{cat}^{ij}∙[E_{i}^{eng}]\leq M∙(1-y^{ij}),1\leq j\leq r,1\leq i\leq p$$ (11)

$$\delta_{e}^{ij}-\beta_{1}∙k_{cat}^{ij}.[E_{i}^{eng}]\leq M∙(1-y^{ij}),1\leq j\leq r,1\leq i\leq p$$ (12)

$$k_{cat}^{ij}∙({\left(\beta_{2}\right)}^{-1}-1)∙[E_{i}^{eng}]\leq \delta_{e}^{ij}\leq k_{cat}^{ij}∙(\beta_{2}-1)∙[E_{i}^{eng}].1\leq j\leq r,1\leq i\leq p$$ (13)

Here, Eq (13) guaranties that the optimized turnover number ($k_{cat}^{ij*}$) is in the allowed range. OKO finds a solution with the minimum number of modified turnover numbers (which is equivalent to the maximum number of unchanged turnover numbers ($\sum_{1\leq j\leq r,1\leq i\leq p}y^{ij}$), while guaranteeing that: (i) the flux towards the objective is at least a given factor, *f*_*pro*_ (≥2) of the maximum product yield and (ii) a certain factor, *α*_*bio*_ (≈1), of optimal growth is achieved. These are enforced by the following constraints:

$$v_{pro}\geq f_{pro}.v_{pro,wt}^{max},$$ (14)

$$v_{bio}\geq \alpha_{bio}.v_{bio,wt}^{opt}.$$ (15)

The combination of the linear objective function of $\underset{v,E,\delta_{e},y}{\max}\sum_{1\leq j\leq r,1\leq i\leq p}y^{ij}$ with the linear constraints in Eqs (2, 3, 7, 8, 9’,10–15) leads to our proposed Mixed-Integer Linear Programming (MILP) formulation of OKO (see Fig 1C).

### Formulation of OKO+

In OKO^+^, we also allow for variation in enzyme abundance. To this end, for each $\left[E_{i}^{eng}\right]$ value (1≤*i*≤*p*), we introduce an interval of $\left((1-\beta_{e})∙[E_{i}^{wt}],(1+\beta_{e})∙[E_{i}^{wt}]\right)$ which, similarly to OKO, defines the range of unchanged values for the abundance. In addition, a binary variable $y_{e}^{i}$ is introduced which takes the value of 1, if the enzyme abundance falls within the interval, and 0, otherwise. Here, *β*_*e*_ (≈10^−1^) is a tuneable parameter that quantifies the deviation from the enzyme abundances in the wild-type model. Based on the following constraints, the binary variable $y_{e}^{i}$ enables us to determine whether the $\left[E_{i}^{eng}\right]$ changes or not:

$$(1-\beta_{e})∙[E_{i}^{wt}]-[E_{i}^{eng}]\leq M∙(1-y_{e}^{i}),1\leq i\leq p$$ (16)

$$-(1+\beta_{e})∙[E_{i}^{wt}]+[E_{i}^{eng}]\leq M∙(1-y_{e}^{i}),1\leq i\leq p$$ (17)

where *M* is a large number (10^6^). In OKO^+^, the constraint in Eq (8) is replaced by the constraints in the Eqs (16) and (17) and the objective is changed to $\underset{v,E,\delta_{e},y}{\max}\sum_{1\leq j\leq r,1\leq i\leq p}y^{ij}+w\sum_{1\leq i\leq p}y_{e}^{i}$, where *w* is the weight reflecting the priority given to the number of changes for enzyme abundance or activity. Given that the primary aim of OKO is to identify modification strategies at the level of catalytic rates, the weight *w* was set to 10 to prioritize minimizing the number of modifications on the enzyme abundance.

### Robustness analysis of enzyme abundance

To assess the precision of the estimated enzyme abundances, for each enzyme abundance we determine the range over which the minimum enzyme usage, from LP1, remains valid. This is done by calculating the minimum and the maximum values the abundance of each enzyme can take at minimum enzyme usage (${sum}_{e}^{*}$) obtained from solving LP1, and can be done by solving the following linear programming problems (LP2):

$$\underset{v,E}{\min}/\max\left[E_{i}\right]\;1\leq i\leq p$$ (18)

s.t

$$\sum_{i=1}^{p}\left[E_{i}\right]={sum}_{e}^{*}$$ (19)

Eqs (2), (3), (4), (5), (6), (7)

Given that the minimum value is usually zero and the maximum is a very small number, we use the log-sum-exp trick [47] to calculate the logarithmic ratio between the maximum and minimum values to deal with the potential numerical instability. As an additional validation step, we compared the variability of enzyme use under both the equality and inequality conditions in Eq 6 and found that the results remained consistent, confirming that our initial assumption did not affect the conclusions.

### OKO and OKO^+^ implementation

OKO and OKO+ both have MILP formulations and are implemented in MATLAB R2022b, available at https://github.com/MonaRazaghi/OKO/. These approaches take as input an ecGEM together with one or a list of compound indices in ecGEM. The output is a list of enzymes with their respective modifications, either on enzyme turn over number or abundance. Both OKO and OKO^+^ use the Gurobi Optimizer v9.5.2 [48] as their solver to efficiently solve the corresponding MILP problems.

OKO includes several tuneable parameters, *α*, *β*_1_ and *β*_2_. To increase the likelihood of finding a solution for the MILP formulation of OKO, the tuneable parameters *α* and *β*_1_ were set to 10^−1^ and 10^−8^, respectively. In addition, the parameter *β*_2_ was set to 10, to avoid large relative changes in *k*_*cat*_ values. In the case of OKO^+^, an additional tunable parameter *β*_*e*_ is included. This parameter was set to 10^−1^ to have reasonable intervals for enzyme abundance.

### Mapping of *k*_*cat*_ values from ecGEMs and DLKcat

The *k*_*cat*_ values in ecGEMs are linked to enzyme-reaction pairs, whereas the predicted *k*_*cat*_ values from the DLKcat approach are linked to enzyme-metabolite pairs. To establish a mapping between the two sets of *k*_*cat*_ values, we first associated the enzymes with their respective EC numbers using the Uniprot database [49]. Enzyme-reaction pairs were then converted to enzyme-metabolite pairs by including all substrates involved in the reaction. Metabolite names from the ecGEMs were mapped to the metabolites present in the DLKcat results using MetaNetX IDs.

## Supporting information

S1 Table The result of applying the OKO method on eciML1515 model for overproducing 41 native metabolites of *E*. *coli*. Columns A-D present the name, KEGG ID, CHEBI ID and formulae of each metabolite, respectively. The maximum production level of each metabolite at optimal growth rate is shown in column E. Column F presents the production level of the metabolite in the engineeered model, where the biomass level is preseneted in cloumn G. The numbers of Kcat that are required to increase and decrease are respectively given in columns H and I, and the sum of fold-changes for Kcats is given in column M. Choline is one metabolite mapped in eciML1515 for which the maximum production is zero in both the wild-type and the engineered model. So, this metabolite is excluded from further analysis.
(XLSX)

S2 Table The result of applying the OKO method on ecYeastGEM_v8.3.4 model for overproducing 49 native metabolites of *S*. *cerevisiae*. Columns A-D present the name, KEGG ID, CHEBI ID and formulae of each metabolite, respectively. The maximum production level of each metabolite at optimal growth rate is shown in column E. Column F presents the production level of the metabolite in the engineeered model, where the biomass level is preseneted in cloumn G. The numbers of Kcat that are required to increase and decrease are respectively given in columns H and I, and the sum of fold-changes for Kcats is given in column M.
(XLSX)

S3 Table Proposed engineering strategies for overproduction of compounds with the least number of modifications required. (XLSX)

S4 Table The log fold change of predicted kcat values for enzymes required for the overproduction of at least three amino acids in eciML1515 model. (XLSX)

S5 Table The log fold change of predicted kcat values for enzymes required for the overproduction of at least three amino acids in ecYeastGEM_v8.3.4 model. (XLSX)

S6 Table Enzymes required reduction in their turnover number for the overproduction of at least one amino acid in eciML1515 model. (XLSX)

S7 Table Enzymes required reduction in their turnover number for the overproduction of at least one amino acid in ecYeastGEM_v8.3.4 model. (XLSX)

S8 Table Comparison of OKO and OptReg. (XLSX)

S9 Table The number of genes and proteins with predicted kcat values in each species, using DLKcat. (XLSX)

S10 Table The number of genes and proteins with predicted kcat values in each species, using TurNup. (XLSX)

S11 Table Enzyme-reaction pair with available k_cat values in the DLKcat results with their corresponding range. (XLSX)

S12 Table Number of enzymes taken from each organism for amino acid modifications, using DLKcat. (XLSX)

S13 Table Enzyme-reaction pair with available k_cat values in the TurNup results with their corresponding range. (XLSX)

S14 Table Number of enzymes taken from each organism for amino acid modifications, using TurNup. (XLSX)
